# Seroprevalence and Associated Factors of 9-Valent Human Papillomavirus (HPV) Types among Men in the Multinational HIM Study

**DOI:** 10.1371/journal.pone.0167173

**Published:** 2016-11-30

**Authors:** Shams Rahman, Christine M. Pierce Campbell, Dana E. Rollison, Wei Wang, Tim Waterboer, Angelika Michel, Michael Pawlita, Luisa L. Villa, Eduardo Lazcano Ponce, Amy R. Borenstein, Anna R. Giuliano

**Affiliations:** 1 Center for Infection Research in Cancer, Moffitt Cancer Center, Tampa, United States of America; 2 Department of Epidemiology and Biostatistics, College of Public Health, University of South Florida, Tampa, United States of America; 3 Molecular Diagnostics of Oncogenic Infections Division; Infection, Inflammation and Cancer Research Program, German Cancer Research Center (DKFZ), Heidelberg, Germany; 4 School of Medicine, University of São Paulo, São Paulo, Brazil; 5 Centro de Investigación en Salud Poblacional, Instituto Nacional de Salud Publica, Cuernavaca, Mexico; Baylor College of Medicine, UNITED STATES

## Abstract

**Background:**

Human papillomavirus (HPV) is one of the most common sexually transmitted infections worldwide. Recently a 9-valent HPV (9vHPV) prophylactic vaccine was licensed. Seroprevalence prior to vaccine dissemination is needed for monitoring vaccine effectiveness over time. Few studies have assessed the seroprevalence of 9vHPV types in men.

**Objectives:**

To investigate the seroprevalence of 9vHPV vaccine types and associated risk factors among men residing in Brazil, Mexico, and the United States.

**Methods:**

Six hundred men were randomly selected from *the HPV Infection in Men* (*HIM) Study*. Archived serum specimens collected at enrollment were tested for antibodies against nine HPV types (6, 11, 16, 18, 31, 33, 45, 52 and 58) using a glutathione S-transferase (GST) L1-based multiplex serologic assay. Socio-demographic, lifestyle and sexual behavior data at enrollment were collected through a questionnaire. Binomial proportions were used to estimate seroprevalence and logistic regression was used to examine factors associated with seropositivity of type-specific and grouped (i.e. 9vHPV, high-risk 9vHPV, low risk 9vHPV, and five-additional) HPV types.

**Results:**

Overall, 28.3% of men were seropositive for at least one of the 9vHPV vaccine types, 14.0% for at least one of the seven high-risk types (16, 18, 31, 33, 45, 52 and 58) and 11.2% for at least one of the five high-risk types (31, 33, 45, 52 and 58) not included in the quadrivalent HPV vaccine, and 17.4% for at least one of the low-risk types (6/11). In multivariate analyses, odds ratios adjusted (AOR) for country of residence, age, marital status, smoking, number of anal sex lifetime partners, compared to men with no anal sex lifetime partners, men with ≥2 partners were more likely to be seropositive for grouped HPV [(9vHPV: AOR 2.52; 95% confidence interval (CI) 1.40–4.54), (high-risk 9vHPV: AOR 2.18; 95%CI: 1.05–4.50) and (low-risk 9vHPV: AOR 2.12; 95%CI: 1.12–4.03)], and individual HPV types 6, 16, 33 and 58 with AORs ranging from 2.19 to 7.36. Compared to men aged 18–30 years, men older than 30 years were significantly more likely to be seropositive for any high-risk 9vHPV, in addition to individual types 18 and 45; and compared to never smokers, current smokers were more likely to be seropositive to 9vHPV, low-risk 9vHPV and HPV 6. In contrast, married men were less likely to be seropositive to any high-risk 9vHPV and individual HPV types 18 and 31 when compared to single men.

**Conclusions:**

These data indicate that exposure to the nine HPV types included in the 9vHPV vaccine is common in men and that seropositivity to 9vHPV vaccine types is associated with older age and the lifetime number of anal sex partners. Nine valent HPV vaccination of males and females has the potential to prevent HPV related diseases and transmission in both sexes.

## Introduction

Human papillomavirus (HPV) is one of the most common sexually transmitted infections (STI) worldwide. Nearly all sexually active adults will be infected with one or more HPV types at some point in their lives [1, 2]. More than 40 HPV types are known to infect the ano-genital region, of which at least 13 are classified as oncogenic [2, 3]. HPV infection is often asymptomatic and transient; however, a small proportion of infections persist and cause benign and malignant diseases in men and women [4, 5]. Oncogenic HPV is a necessary cause of cervical cancer and nearly all cervical, approximately 50% of vulvar, and 65% of vaginal cancers, are caused by oncogenic HPV [1, 6]. HPV also causes approximately 40–50% of penile, 85% of anal, 60–70% of oropharyngeal, and 10% of laryngeal cancers [2, 7–10]. Non-oncogenic HPV types cause genital warts and other benign lesions of epithelial tissues [11].

In the United States (U.S.), the bivalent and 4-valent HPV vaccines are recommended for routine vaccination for girls at age 11 or 12 years, females age 13 through 26 years, and males age 13 through 21 years if they have not yet received the HPV vaccine [12]. Recently, the Advisory Committee on Immunization Practices (ACIP) added the 9-valent HPV vaccine (9vHPV) to the vaccine recommendations [13]. In 2006, the 4-valent HPV vaccine (4vHPV) was approved for use among women in Brazil [14], and the vaccine was authorized in Mexico in 2008 [15]. HPV vaccine recommendation for men has yet to emerge in these two countries. The World Health Organization (WHO) issues periodic position papers on vaccine implementation. These papers are reviewed and endorsed by the WHO Strategic Advisory Group of Experts (SAGE) on vaccines and immunization before dissemination to Member States. The first WHO position paper on 4vHPV vaccine was published in April of 2009 [16]. However, this position paper did not recommend HPV vaccination of males, arguing that limited public health resources should be directed toward achieving high coverage (>70%) of HPV vaccination among the primary target population (young adolescent girls) to prevent cervical cancer. In the revised position paper, dated October 2014, WHO upgraded its HPV vaccine recommendations and included secondary target populations (older adolescent females), but once again did not recommend HPV vaccination of males, citing the same a forth mentioned reason [17]. Information on the natural history of HPV infection and serology in men prior to vaccine dissemination is needed to monitor the effectiveness of the vaccine over time.

Although a number of studies have reported seroprevalences of HPV types 6, 11, 16 and 18 in men from different countries [18–23], few have examined other HPV types (e.g. 31, 33, 45, 52 and 58) included in the 9vHPV vaccine in the general population. One study included only men who have sex with men (MSM) [24], another study included men only from the U.S. with an age range of 14–59 years [25], and two studies using the same study population in the Netherlands examined only the seven high-risk types in the 9vHPV vaccine [26, 27]. Previously, we reported seroprevalences for the 4-valent HPV vaccine types (6, 11, 16 and 18) among men [20]. In this study, we extended our analysis to estimate the seroprevalence of 9vHPV vaccine types and investigated factors associated with the seropositivity in men from three countries (Brazil, Mexico, and U.S.) across a broad age range to report data for the entire adult lifespan and old age.

## Methods

### Study Population

Study participants included a sub-cohort of 600 men randomly selected from *the HPV Infection in Men (HIM) Study*. Simple random sampling method was used to select the sub-cohort because performing serology testing for the entire parent cohort was not feasible and cost-effective. These 600 men did not report a history of receipt of an HPV vaccine.The *HIM Study* uses a prospective cohort design to examine the natural history of HPV infections among men in three countries. Detailed descriptions of the study population and procedures have been published elsewhere [28, 29]. Briefly, between July 2005 and September 2009, over 4000 men aged 18–70 years at baseline were recruited from Tampa, Florida, Sao Paulo, Brazil, and Cuernavaca, Mexico. Eligibility criteria included no previous history of penile cancer, anal cancer, ano-genital warts and HIV; no current history or treatment for STIs; no current discharges from the penis or burning sensation during urination; and no history of prior or current participation in HPV vaccine trails. Participants were followed every six months for a median of four years. At enrollment and each study visit, participants completed a computer-assisted self-interviewed questionnaire, provided blood and urine specimens, and underwent a clinical examination. A total of 3,695 *HIM Study* participants who provided serum specimens and completed the questionnaire at enrollment were eligible to be included in the current study. All participants provided written informed consent. This study was approved by the Institutional Review Boards of the University of South Florida (Tampa, FL, USA), the Ludwig Institute for Cancer Research (Sao Paulo, Brazil), the Centro de Referencia e Treinamento em Doencas Sexualmente Transmissiveis e AIDS (Sao Paulo, Brazil), and the Instituto Nacional de Salud Publica de Mexico (Cuernavaca, Mexico).

### Specimens and Data Collection

At the enrollment visit, *the HIM Study* participants provided detailed information on sociodemographic characteristics, smoking habits, alcohol consumption, medical history, and an extensive sexual behaviors. Archived enrollment serum specimens from 600 participants, collected between July 2005 and September 2009, were tested for seroreactivity to L1 major capsid proteins of the 9vHPV vaccine types (6, 11, 16, 18, 31, 33, 45, 52 and 58). The antibody detection method was based on a glutathione S-transferase (GST) capture enzyme-linked immunosorbent assay (ELISA), in combination with fluorescent bead technology previously described [30, 31]. To define seropositivity for each of the 9vHPV vaccine types, type-specific cut-off values measured in median fluorescence intensity [MFI] units were applied, as previously described [32].

### Statistical Analysis

Participants in the randomly selected sub-cohort (n = 600) were compared to the full *HIM* cohort (>4,000 men) on all baseline socio-demographic and sexual behavioral characteristics listed in Table 1 using Chi-square test for categorical variables. Type-specific seroprevalence was calculated as the proportion of men who tested positive for a given HPV type. Four additional categories of ‘grouped HPV’ (i.e. 9vHPV, high-risk 9vHPV, 5-additional HPV, and low-risk 9vHPV) were also used as dependent variables in the analyses. ‘9vHPV’ seroprevalence was defined as the proportion of men who were seropositive for at least one of the nine vaccine types. ‘High-risk 9vHPV’ included men who were seropositive for at least one of the seven oncogenic types (i.e. 16, 18, 31, 33, 45, 52 and 58), ‘5-additional HPV’ included men who were seropositive for at least one of the five oncogenic types not included in the quadrivalent HPV vaccine (i.e. 31, 33, 45, 52 and 58), and ‘low-risk 9vHPV’ included men who were seropositive for HPV types 6 or 11. Two subjects with inadequate serology results were excluded from all analyses resulting in a final sample size of 598 men. Enrollment sociodemographic and behavioral factors were compared between seropositive and seronegative men with high- and low-risk 9vHPV using Chi-square test (Table 1). Seroprevalence estimates were calculated for each HPV type, by country and age group, and compared using Chi-square and Fisher exact tests. The Holm–Bonferroni correction was used to adjust for post-hoc pairwise comparisons of seroprevalence estimates by country. To examine associations between individual and grouped HPV seropositivity and potential risk factors, logistic regression was used and odds ratios (ORs) and their 95% confidence intervals (CI) were estimated. Factors listed in Table 1 were considered for inclusion in the multivariate logistic regression models. Variables were selected using backward stepwise elimination with significance at p≤0.1. Country and age were forced into the model due to the study design. To assess the individual contribution of each variable retained in the model at p≤0.1, the likelihood ratio test at p<0.05 was performed. The final multivariate models for 9vHPV and low-risk 9vHPV included country, age, smoking, and the lifetime number of male anal sex partners, and in the final model for high-risk 9vHPV, marital status was retained and smoking status was dropped. Multivariate models for individual high-risk HPV types 16, 18, 31, 33, 45, 52 and 58 were adjusted for the same factors as the grouped high-risk 9vHPV model, and multivariate models for individual low-risk HPV types 6 and 11 were adjusted for the same factors as the grouped low-risk 9vHPV model. All analyses were performed in SAS 9.3.

**Table 1:** Participant characteristics by seropositivity to high-risk and low-risk types in 9vHPV vaccine.

	High-risk 9vHPV (16, 18, 31, 33, 45, 52, 58)					Low-risk 9vHPV (6, 11)				
	Seronegative		Seropositive		P-value^a^	Seronegative		Seropositive		P-value^a^
Characteristic	N	%	N	%		N	%	N	%	
**Country**										
USA	165	89.7	19	10.3	0.173	165	89.7	19	10.3	**<0.001**
Brazil	171	85.5	29	14.5		145	72.5	55	27.5	
Mexico	178	83.2	36	16.8		184	86.0	30	14.0	
**Age, Years**										
18–30	233	90.0	26	10.0	**0.018**	213	82.2	46	17.8	0.976
31–44	216	84.4	40	15.6		212	82.8	44	17.2	
45–73	65	78.3	18	21.7		69	83.1	14	16.9	
**Race**										
White	225	83.6	44	16.4	0.248	213	79.2	56	20.8	0.229
Black	72	92.3	6	7.7		67	85.9	11	14.1	
Asian	15	88.2	2	11.8		15	88.2	2	11.8	
American Indian/Alaska Native	9	100.0	0	0.0		6	66.7	3	33.3	
Other	188	86.2	30	13.8		186	85.3	32	14.7	
**Ethnicity**										
Hispanic	240	85.1	42	14.9	0.532	237	84.0	45	16.0	0.383
Non-Hispanic	265	86.9	40	13.1		248	81.3	57	18.7	
**Education, Years**										
12 or less	242	84.3	45	15.7	0.104	233	81.2	54	18.8	0.572
13–15	145	91.2	14	8.8		131	82.4	28	17.6	
16 or more	126	84.6	23	15.4		127	85.2	22	14.8	
**Marital Status**										
Single/never married	224	86.5	35	13.5	0.140	207	79.9	52	20.1	0.232
Married/cohabitating	243	87.4	35	12.6		234	84.2	44	15.8	
Divorced/separated/widowed	45	77.6	13	22.4		51	87.9	7	12.1	
**Smoking Status**										
Never	292	86.1	47	13.9	0.909	289	85.3	50	14.7	0.104
Former	98	86.7	15	13.3		92	81.4	21	18.6	
Current	124	84.9	22	15.1		113	77.4	33	22.6	
**Alcohol, No. Drinks/Month**										
0	123	87.2	18	12.8	0.818	110	78.0	31	22.0	0.124
1–30	238	85.3	41	14.7		239	85.7	40	14.3	
31–60	48	82.8	10	17.2		44	75.9	14	24.1	
61 or more	96	87.3	14	12.7		92	83.6	18	16.4	
**Circumcision**										
No	332	85.3	57	14.7	0.561	312	80.2	77	19.8	**0.034**
Yes	182	87.1	27	12.9		182	87.1	27	12.9	
**Sexual Orientation**										
MSW	457	87.2	67	12.8	**0.011**	445	84.9	79	15.1	**0.001**
MSM	39	76.5	12	23.5		34	66.7	17	33.3	
MSWM	10	66.7	5	33.3		8	53.3	7	46.7	
**No. of Female LTP**										
0	53	88.3	7	11.7	0.663	45	75.0	15	25.0	0.372
1–3	105	83.3	21	16.7		106	84.1	20	15.9	
4–18	214	87.0	32	13.0		201	81.7	45	18.3	
19 or more	111	88.1	15	11.9		107	84.9	19	15.1	
**No. of Male Anal LTP**										
0	440	86.8	67	13.2	**0.003**	431	85.0	76	15.0	**0.001**
1	28	100.0	0	0.0		21	75.0	7	25.0	
2 or more	43	74.1	15	25.9		38	65.5	20	34.5	
**No. of female sex partners in the past 6 months among those reporting ever having a male sex partner**										
0	93	80.9	22	19.1	0.096	101	87.8	14	12.2	0.111
1	215	89.2	26	10.8		205	85.1	36	14.9	
2 or more	138	85.2	24	14.8		128	79.0	34	21.0	
**No. of male sex partners in the past 6 months among those reporting ever having a male sex partner**										
0	48	87.3	7	12.7	**0.021**	41	74.6	14	25.4	0.256
1	12	92.3	1	7.7		7	53.8	6	46.2	
2 or more	13	61.9	8	38.1		13	61.9	8	38.1	

Note: LTP = lifetime partners, MSW = men who have sex with women; MSM = men who have sex with men; MSWM = men who have sex with women and men.

^a^ Chi-square test was used to calculate p-values. When 1 or more cells had an expected frequency of 5 or less then Fisher’s exact test was used. Missing values were not included in p-value calculations. Significant p-value is marked in bold.

## Results

Participants in the randomly selected sub-cohort (n = 600) were similar to the full *HIM* cohort (>4,000 men) on all baseline sociodemographic and sexual behavioral characteristics listed in Table 1 (p>0.05). The study included 184 participants from the U.S., 200 from Brazil, and 214 from Mexico. None of the 600 men reported a history of HPV vaccine. Participant characteristics by seropositivity to grouped high- and low-risk 9vHPV categories are presented in Table 1. Significant differences by age, sexual orientation, lifetime number of anal sex partners (LTP), and recent male anal sex partners were observed for seropositivity to high-risk 9vHPV (p<0.05). Mid-adult men (35–44 years) and older men (45–73 years), men who have sex with men (MSM), men who have sex with men and women (MSMW), men with ≥2 male anal sex partners LTP, and men with ≥2 recent anal sex partners were more likely to be seropositive for high-risk 9vHPV (p<0.05). Significant differences in seropositivity to low-risk 9vHPV were observed by country, circumcision status, sexual orientation, and number of male anal sex LTP (p<0.05).

**Fig 1:**
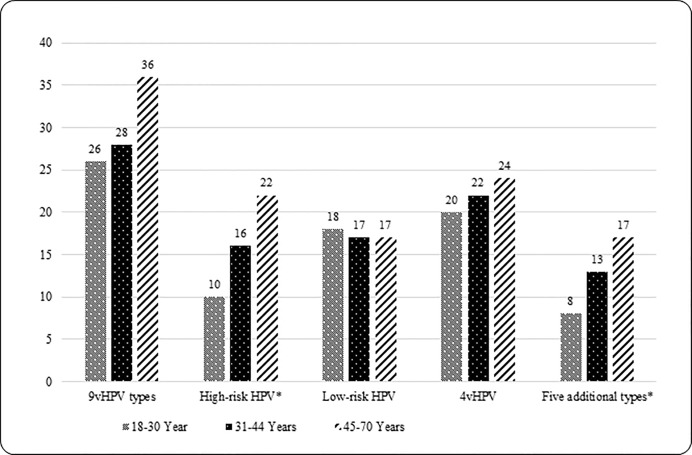
Grouped HPV seroprevalence of nine vaccine types by age group. 9vHPV category included nine vaccine types (6, 11, 16, 18, 31, 33, 45, 52, and 58). ‘9vHPV’ variable was created, if a man was positive for one or more of the nine vaccine types then the ‘9vHPV’variable was seropositive otherwise seronegative. 4vHPV category included seropositivity to at least one of the four types of HPV (6, 11, 16 and 18). High-risk 9vHPV category included seropositivity to at least one of the seven types of HPV (16, 18, 31, 33, 45, 52 and 58). Low-risk 9vHPV category included seropositivity to at least one of the two types of HPV (6 and 11). Significant p-value <0.05 is marked with ‘*’. P-value was obtained from Cochran-Armitage Trend Test. P_trend_ <0.05 for high-risk and five additional types categories.

**Fig 2:**
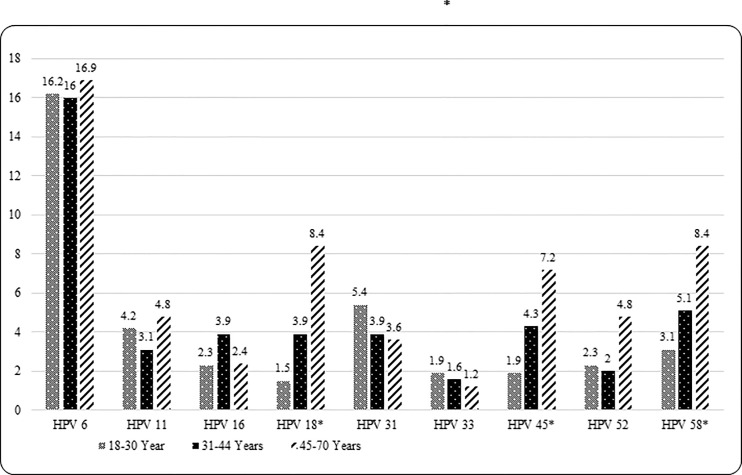
Type-specific seroprevalence of nine HPV vaccine types by age group. Significant p-value <0.05 is marked with ‘*’. P-value was obtained from Cochran-Armitage Trend Test. P_trend_ <0.05 for HPV types 18, 45 and 58.

Grouped and individual type HPV seroprevalence estimates are presented in Table 2. Overall, 28.3% of men were seropositive for ≥1 9vHPV type, 14.0% for ≥1 high-risk 9vHPV types, 11.2% for ≥1 of the five additional types, and 17.4% for low-risk 9vHPV types 6 and/or 11. Type-specific seropositivity for five individual HPV types was as follows: HPV 31 (4.5%), HPV 33 (1.7%), HPV 45 (3.7%), HPV 52 (2.5%), and HPV 58 (4.7%). Seropositivity for 9vHPV was statistically significantly different by country [Brazil (36.5%), Mexico (28.5%) and U.S. (19.0%); overall p<0.001] and remained significant only for Brazil vs. U.S. (p<0.001) after pairwise comparisons. No significant differences in the seroprevalence of grouped high-risk 9vHPV types was observed by country [Brazil (14.5%), Mexico (16.8%) and U.S. (10.3%); overall p = 0.173]. Seroprevalence of the five-additional types also did not vary significantly by country [Brazil (10.5%) and Mexico (13.6) and U.S. (9.2%); overall p = 0.368]. Seropositivity to low-risk 9vHPV was statistically significantly different by country [Brazil (27.5%), Mexico (14.0%) and U.S. (10.3%); overall p<0.001] and remained significant for Brazil vs. U.S. (p<0.001) and Brazil vs. Mexico (p<0.001) after pairwise comparisons. Seroprevalence of individual HPV types 6 and 16 significantly differed by country [Brazil (25.5%) and (5.5%), Mexico (13.6%) and (1.9%) and U.S. (9.2%) and (1.6%), respectively; overall p-values<0.05]. After pair wise comparisons, significant difference remained for Brazil vs. U.S. (p<0.001) and Brazil vs. Mexico (p = 0.004) only for HPV 6. Seroprevalence was highest for HPV 6 in all three countries [Brazil (25.5%), Mexico (13.6%) and U.S. (9.2%)]. Figs 1 and 2 show grouped and type-specific HPV seroprevalence by age group. A significant positive trend (p_trend_ <0.05) for seropositivity with increasing age was observed for grouped high-risk 9vHPV, grouped five-additional types, and some individual HPV types (i.e. 18, 45 and 58).

**Table 2:** Grouped and type-specific seroprevalence of 9vHPV types among men from Brazil, Mexico and the United States

HPV Type	Overall (N = 598)	U.S. (N = 184)	Brazil (N = 200)	Mexico (N = 214)	P-value^f^
	%	%	%	%	
HPV 6	16.2	9.2	25.5	13.6	**<0.001**
HPV 11	3.8	2.2	6.0	3.3	0.129
HPV 16	3.0	1.6	5.5	1.9	**0.041**
HPV 18	3.5	1.1	4.0	5.1	0.082
HPV 31	4.5	3.8	4.0	5.6	0.628
HPV 33	1.7	1.6	2.0	1.4	0.892
HPV 45	3.7	1.6	5.0	4.2	0.189
HPV 52	2.5	3.3	1.5	2.8	0.513
HPV 58	4.7	6.5	2.0	5.6	0.081
9vHPV^a^	28.3	19.0	36.5	28.5	**0.001**
High-risk (16, 18, 31, 33, 45, 52, 58)^b^	14.0	10.3	14.5	16.8	0.173
Five additional types (31, 33, 45, 52, 58)^c^	11.2	9.2	10.5	13.6	0.368
Low-risk (6, 11)^d^	17.4	10.3	27.5	14.0	**<0.001**
4vHPV types (6, 11, 16, 18)^e^	21.2	12.5	32.0	18.7	**<0.001**

^a.^ Grouped 9vHPV category included nine vaccine types (6, 11, 16, 18, 31, 33, 45, 52 and 58). ‘9vHPV’ variable was created, if a man was positive for one or more of the 9 types in the vaccine then the ‘9vHPV’ variable was seropositive otherwise seronegative.

^b.^ Grouped high-risk 9vHPV category included seropositivity to at least one of the seven types of HPV (16, 18, 31, 33, 45, 52 and 58).

^c.^ Grouped five additional category included seropositivity to at least of the five types of HPV (31, 33, 45, 52 and 58).

^d.^ Grouped low-risk 9vHPV category included seropositivity to at least one of the two types of HPV (6 and 11).

^e.^ Grouped 4vHPV category included seropositivity to at least one of the four types of HPV (6, 11, 16 and 18).

^f.^ P-values were calculated by Chi-square test, and Fisher exact test was used when 1 or more cell had an expected frequency of 5 or less. Significant p-value is marked in bold.

Factors associated with seropositivity of grouped and specific individual HPV types are presented in Tables 3–5. In multivariate analyses, adjusting for all other variables in the table, lifetime number of anal sex partners, was significantly associated with seropositivity for grouped and specific individual HPV types (6, 16, 33 and 58). Compared to men with no reported anal sex, men with ≥2 anal sex LTP were more likely to be seropositive for grouped HPV [(9vHPV: adjusted odds ratio (AOR) 2.52; 95%CI: 1.40–4.54), (high-risk 9vHPV: AOR 2.18; 95%CI: 1.05–4.50) and (low-risk 9vHPV: AOR 2.12; 95%CI: 1.12–4.03)] respectively. Similarly, men with ≥2 anal sex LTP were significantly more likely to be seropositive for individual HPV types 6, 16, 33 and 58, with AORs ranging from 2.19 to 7.36. Increasing age was significantly positively associated with seropositivity for high-risk 9vHPV and HPV types 18 and 45. Compared to men aged 18–30 years, men aged 31–44 years, and men aged 45–73 were more likely to be seropositive for high-risk 9vHPV [(AOR 1.95; 95%CI: 1.03–3.70) and (AOR 2.75; 95%CI: 1.23–6.15), respectively] and HPV 18 [(AOR 4.60; 95%CI: 1.24–17.10) and (AOR 14.47; 95%CI: 3.24–64.68) respectively]. Compared to men aged 18–30 years, men aged 45–73 years were more likely to be seropositive for HPV 45 (AOR 5.43; 95%CI: 1.27–23.25). Country was significantly associated with seropositivity for grouped and individual HPV types. Compared to men from the U.S., men from Brazil were more likely to be seropositive for 9vHPV (AOR 2.18; 95%CI: 1.31–3.60), low-risk 9vHPV (AOR 3.13; 95%CI: 1.71–5.75), HPV 6 (AOR 3.15; 95% CI: 1.67–5.93), HPV 11 (AOR 3.43; 95%CI: 1.04–11.35), and less likely to be seropositive to HPV 58 (AOR 0.12; 95%CI: 0.03–0.53). Similarly, compared to men from the U.S., men from Mexico were more likely to be seropositive for 9vHPV (AOR 1.66; 95%CI: 1.02–2.71), high-risk 9vHPV (AOR 2.24; 95%CI: 1.15–4.40) and HPV 18 (AOR 7.91; 95%CI: 1.51–41.34). Compared to never smokers, current smokers were more likely to be seropositive to 9v-HPV, low-risk 9vHPV and HPV 6. In contrast, compared to men who reported being single/never married, married/cohabiting men were less likely to be seropositive to high-risk 9vHPV, HPV types 18 and 31 (Tables 3–5).

**Table 3:** Factors independently associated with seropositivity of nine vaccine type HPV in men from Brazil, Mexico and the United States

	9vHPV^a^		High-risk 9vHPV^b^		Low-risk 9vHPV^c^		HPV 6	
Characteristics	OR (95%CI)	AOR (95%CI)^d^	OR (95%CI)	AOR (95%CI)^d^	OR (95%CI)	AOR (95%CI)^d^	OR (95%CI)	AOR (95%CI)^d^
**Country**								
U.S.	1.00	1.00	1.00	1.00	1.00	1.00	1.00	1.00
Brazil	**2.45**	**2.18 (1.31–3.60)**	1.47	1.17 (0.59–2.35)	**3.29**	**3.13 (1.71–5.75)**	**3.36**	**3.15 (1.67–5.93)**
Mexico	**1.70**	**1.66 (1.02–2.71)**	1.76	**2.24 (1.15–4.40)**	1.42	1.39 (0.74–2.62)	1.54	1.48 (0.77–2.86)
**Age, Years**								
18–30	1.00	1.00	1.00	1.00	1.00	1.00	1.00	1.00
31–44	1.12	0.95 (0.63–1.43)	1.66	**1.95 (1.03–3.70)**	0.96	0.78 (0.48–1.26)	0.99	0.79 (0.48–1.30)
45–73	1.62	1.43 (0.81–2.51)	**2.48**	**2.75 (1.23–6.15)**	0.94	0.83 (0.41–1.68)	1.05	0.92 (0.45–1.89)
**Marital Status**								
Single or never married	—	—	1.00	1.00	—	—	—	—
Married/cohabitating	—	—	1.02	**0.50 (0.26–0.99)**	—	—	—	—
Divorced/separated/widowed	—	—	1.15	1.18 (0.52–2.70)	—	—	—	—
**Smoking Status**								
Never	1.00	1.00	—	—	1.00	1.00	1.00	1.00
Current	1.51	**1.57 (1.01–2.44)**	—	—	**1.69**	**1.96 (1.17–3.29)**	**1.83**	**2.12 (1.25–3.61)**
Former	1.14	1.14 (0.69–1.89)	—	—	1.32	1.52 (0.84–2.77)	1.41	1.58 (0.85–2.91)
**No. of Male Anal Sex LTP**								
0	1.00	1.00	1.00	1.00	1.00	1.00	1.00	1.00
1	0.97	0.78 (0.32–1.92)	NE	NE	1.89	1.37 (0.54–3.44)	2.08	1.52 (0.6–3.83)
2 or more	**3.11**	**2.52 (1.40–4.54)**	**2.29**	**2.18 (1.05–4.50)**	**2.98**	**2.12 (1.12–4.03)**	**3.04**	**2.19 (1.14–4.21)**

OR = unadjusted odds ratio; AOR = adjusted odds ratio; CI = 95% confidence interval; LTP = lifetime partners

‘—‘ variable not included in the final adjusted model

^a.^ This category included seropositivity to 1 or more of the nine vaccine types (6, 11, 16, 18, 31, 33, 45, 52, 58)

^b^. This category included seropositivity to 1 or more of the high-risk types in the vaccine (16, 18, 31, 33, 45, 52, 58)

^c.^ This category included seropositivity to 1 or more of the low-risk types in the vaccine (16, 18)

^d.^ Final adjusted models were estimated using backward stepwise elimination method with a significance level of p-value ≤ 0.1 for retention. Odds ratios were adjusted for all other variables in the column.

**Table 4:** Factors independently associated with seropositivity of nine vaccine type HPV in men from Brazil, Mexico and the United States

	HPV 11		HPV 16		HPV 18		HPV 31	
Characteristics	OR (95%CI)	AOR (95%CI)^d^	OR (95%CI)	AOR (95%CI)^d^	OR (95%CI)	AOR (95%CI)^d^	OR (95%CI)	AOR (95%CI)^d^
**Country**								
U.S.	1.00	1.00	1.00	1.00	1.00	1.00	1.00	1.00
Brazil	2.87	**3.43 (1.04–11.35)**	3.51	1.71 (0.4–7.39)	3.79	3.22 (0.61–16.97)	1.05	1.08 (0.34–3.42)
Mexico	1.52	1.66 (0.47–5.91)	1.15	1.43 (0.29–7.10)	**4.93**	**7.91 (1.51–41.34)**	1.50	2.65 (0.93–7.58)
**Age, Years**								
18–30	1.00	1.00	1.00	1.00	1.00	1.00	1.00	1.00
31–44	0.73	0.62 (0.24–1.60)	1.71	1.52 (0.42–5.42)	2.59	**4.60 (1.24–17.1)**	0.71	1.08 (0.39–2.98)
45–73	1.14	0.98 (0.29–3.35)	1.04	0.59 (0.08–4.26)	**5.87**	**14.47 (3.24–64.68)**	0.66	1.04 (0.24–4.52)
**Marital Status**								
Single or never married	—	—	1.00	1.00	1.00	1.00	1.00	1.00
Married/cohabitating	—	—	0.76	0.66 (0.17–2.56)	0.89	**0.17 (0.05–0.56)**	0.60	**0.32 (0.10–0.99)**
Divorced/separated/widowed	—	—	1.31	3.58 (0.84–15.19)	0.37	0.11 (0.01–1.00)	0.37	0.82 (0.20–3.42)
**Smoking Status**								
Never	1.00	1.00	—	—	—	—	—	—
Current	1.50	1.73 (0.65–4.65)	—	—	—	—	—	—
Former	1.38	1.57 (0.51–4.84)	—	—	—	—	—	—
**No. of Male Anal Sex LTP**								
0	1.00	1.00	1.00	1.00	1.00	1.00	1.00	1.00
1	NE	NE	NE	NE	NE	NE	NE	NE
2 or more	1.90	1.34 (0.41–4.41)	**8.85**	**7.33 (2.22–24.17)**	0.50	2.48 (0.78–7.93)	1.63	1.72 (0.51–5.88)

OR = unadjusted odds ratio; AOR = adjusted odds ratio; CI = 95% confidence interval; LTP = lifetime partners

‘—‘ variable not included in the final adjusted model

d. Final adjusted models were estimated using backward stepwise elimination method with a significance level of p-value ≤ 0.1 for retention. Odds ratios were adjusted for all other variables in the column.

**Table 5:** Factors independently associated with seropositivity of nine vaccine type HPV in men from Brazil, Mexico and the United States

	HPV 33		HPV 45		HPV 52		HPV 58	
Characteristics	OR (95%CI)	AOR (95%CI)^d^	OR (95%CI)	AOR (95%CI)^d^	OR (95%CI)	AOR (95%CI)^d^	OR (95%CI)	AOR (95%CI)^d^
**Country**								
U.S.	1.00	1.00	1.00	1.00	1.00	1.00	1.00	
Brazil	1.23	0.8 (0.14–4.60)	3.17	2.52 (0.62–10.28)	0.45	0.30 (0.06–1.51)	**0.29**	**0.12 (0.03–0.53)**
Mexico	0.86	1.47 (0.26–8.26)	2.65	3.05 (0.72–12.87)	0.86	0.99 (0.27–3.67)	0.85	0.87 (0.33–2.29)
**Age, Years**								
18–30	1.00	1.00	1.00	1.00	1.00	1.00	1.00	
31–44	0.81	0.83 (0.17–4.03)	2.28	3.14 (0.94–10.51)	0.84	0.63 (0.15–2.57)	1.68	1.82 (0.61–5.44)
45–73	0.62	0.48 (0.04–6.05)	**3.96**	5.43 (1.27–23.25)	2.14	1.22 (0.25–5.90)	**2.89**	2.90 (0.81–10.47)
**Marital Status**								
Single or never married	1.00	1.00	1.00	1.00	1.00	1.00	1.00	1.00
Married/cohabitating	0.20	0.36 (0.06–2.35)	1.00	0.38 (0.12–1.17)	0.69	1.35 (0.29–6.21)	0.98	0.83 (0.26–2.62)
Divorced/separated/widowed	1.04	2.05 (0.29–14.39)	0.37	0.18 (0.02–1.57)	0.76	4.72 (0.90–24.77)	1.93	1.80 (0.47–6.81)
**Smoking Status**								
Never	—	—	—	—	—	—	—	—
Current	—	—	—	—	—	—	—	—
Former	—	—	—	—	—	—	—	—
**No. of Male Anal Sex LTP**								
0	1.00	1.00	1.00	1.00	1.00	1.00	1.00	1.00
1	NE	NE	NE	NE	NE	NE	NE	NE
2 or more	**6.19**	**7.36 (1.56–34.77)**	**2.90**	1.9 (0.59–6.14)	2.25	4.03 (0.89–18.28)	1.99	**4.49 (1.36–14.8)**

OR = unadjusted odds ratio; AOR = adjusted odds ratio; CI = 95% confidence interval; LTP = lifetime partners

‘—‘ variable not included in the final adjusted model

d. Final adjusted models were estimated using backward stepwise elimination method with a significance level of p-value ≤ 0.1 for retention. Odds ratios were adjusted for all other variables in the column.

## Discussion

Previously, we reported seroprevalence of the 4-valent HPV vaccine types (6, 11, 16 and 18) [20]. In this study, we extended our analysis to include five additional HPV types (31, 33, 45, 52 and 58) in the 9vHPV vaccine. Additionally, we investigated factors associated with 9vHPV seropositivity in 598 men recruited from Brazil, Mexico and the U.S. across a broad age range (18–73 years). Participants in the parent cohort (*the HIM Study*) were recruited between July 2005 and September 2009. The 4-valent HPV vaccine was approved in September of 2009, and the 9-valent HPV vaccine in February of 2015, for use among men. Out of the 600 men analyzed in the current manuscript, 594 were recruited before September 2009. Only 6 men were recruited in September of 2009. Furthermore, during the baseline and follow up visits, study participants were also asked about their HPV vaccination status. None of the 600 men reported a history of HPV vaccination. Therefore, positive serologic results for all HPV types examined in this report represent immune response to natural infections. Over 28.3% of men were seropositive for ≥1, 9vHPV vaccine types, and 11.2% for ≥1 of the five additional high-risk types. Type specific seropositivity for five additional types was as follows: [HPV 31 (4.5%), 33 (1.7%), 45 (3.7%), 52 (2.5%), and 58 (4.7%)]. Age, lifetime number of male anal sex partners, and smoking were positively associated with seropositivity of grouped and individual HPV types. A recent study [25] in U.S. used competitive Luminex Immunoassay (cLIA) and estimated seroprevalence of 9vHPV vaccine types among men using the National Health and Nutrition Examination Survey (NHANES) data. The seroprevalence estimates for 9vHPV, and 5-additional types in our study were higher than the NHANES study [(28.3% vs. 19.4%) and (11.2% vs. 6.6%) respectively]. The type-specific seroprevalence estimates of the five additional types were also slightly higher in our study [HPV 31 (4.5% vs. 2.3%), 33 (1.7% vs. 1.6%), 45 (3.7% vs. 0.7%), 52 (2.5% vs. 1.3%), and 58 (4.7% vs. 1.5%). Another study [27] estimated the seroprevalence of five additional types in the Netherlands using virus-like particles (VLP) based serologic assay and reported slightly higher seroprevalence estimates than our study for HPV types 33 (1.7% vs. 6.0%), 45 (3.7% vs. 6.8%) and 52 (2.5% vs.5.2%); and slightly lower estimates for types 31 (4.5% vs. 2.5%) and 58 (4.7% vs. 3.7%). Some of this variation could be partially explained by the differences in serologic assays used, study population, and age distribution across studies.

Seroprevalence estimates of grouped and individual 4-valent HPV vaccine types (11, 16 and 18) in our study were considerably lower than the estimates we had previously reported using VLP-based serologic assay, (HPV 11 [3.8% in current study vs. 17.3% in previous study], HPV 16 [3.0 vs. 11.2%], HPV 18 [3.5% vs. 5.8%]) [20]. Whereas important demographic and sexual behavior characteristics such as age, marital status, education, and lifetime number of anal sex partners were similar in both studies, except for country of residence. Compared to previous study, current study had slightly fewer men from U.S. (31% vs. 37%) and slightly higher men from Brazil (33 vs. 30%). Since men from U.S. are less likely, and men from Brazil are more likely, to be seropositive for 4-valent HPV types as shown in both studies, this difference by country of residence is not likely to underestimate the seroprevalence estimates in the current study compared to previously reported. We believe that this variation is due to differences in the serologic assays used in current and previous study, and that the GST L1-based multiplex serologic assay underestimated seroprevalence estimates for 4vHPV types in the current study, except for HPV type 6. Nevertheless, both studies consistently reported that men with multiple lifetime number of anal sex partner, older age, single/never married men, and Brazilians were more likely to be seropositive for 4-valent HPV types.

The positive association of increasing age and high-risk 9vHPV in this study is consistent with previous serology studies in men [18, 20, 33, 34], and this may reflect the fact that exposure to HPV and antibodies as a response accumulate over time. The positive association of the increasing number of male anal sex LTP with the seropositivity of HPV is also consistent with previous studies [20, 35] and is likely to be explained by i) the increased probability of exposure to HPV types due to multiple sex partners, ii) the highest DNA prevalence of HPV infection and persistence in the anal canal of MSM, and iii) the anatomic site-specific immune response. Studies have shown that HPV infection is common in the anal canal, and its prevalence is higher among MSM than men who have sex with women (MSW) [35–37]. Recently, emerging evidence showed that HPV infection persists longer in the anal canal of MSM [38] which may explain the highest incidence of HPV-related anal cancers in MSM [39]. Also, it is suggested that the viral antigen is more efficiently detected by the immune system in the mucosal epithelium. Therefore, mucosal epithelium (e.g. anal canal) is more likely to induce immune response to HPV infections compared to keratinized epithelium (e.g. penile skin) [39–41]. As a result, MSM are more likely to have incident, prevalent, and persistent HPV infections, and potentially may induce greater immune response because of the anatomic location of their infection compared to other sub-groups (e.g. MSW) resulting in high seroprevalence. Current smokers were more likely to be seropositive to low-risk 9vHPV (AOR 1.96; 95%CI: 1.17–3.29). Syrjanen et al. [42] and Dunne et al. [18] also reported a two-fold increased risk of low-risk 9vHPV (6/11) seropositivity for current male smokers. However, Liu et al. [25] did not find an association between smoking and low-risk 9vHPV. In contrast, married/cohabiting men were less likely to be seropositive for high-risk 9vHPV compared to single/never married men. Similar associations have also been reported by other studies previously [27, 43, 44]. This association is likely to be partially explained that single men might have more opportunities of meeting new sex partners compared to married men, therefore, they might have a high probability of exposure to different HPV types.

Inclusion of men from three countries across a broad range of ages, detailed information on demographic and sexual history, and the estimation of prevalence based on type-specific antibodies against L1 HPV capsid protein in a single laboratory with one protocol are the strengths of this study. Some limitations should be considered when interpreting the results. This study was a cross-sectional analysis which utilized serology and risk factor data collected at enrollment. HPV prevalence based on antibody response may be affected by waning of antibodies over time and by the seroconversion rate since all infected individuals do not produce an immune response to HPV infections; therefore, prevalence and cumulative exposure of HPV might be underestimated. Seropositivity in our study was measured by GST-based multiplex serologic assay and may not be comparable to estimates measured using other assays. Risk factor data was self-reported. Due to social desirability and recall biases, lifetime number of sex partners may be under- or over-reported. However, the use of computer-assisted self-interviews for sexual behavior data may have minimized the social desirability bias. Despite these limitations, this study provides important data on the distribution of 9-valent HPV vaccine types in a sample of men from three countries. In summary, more than a quarter of men were seropositive for at least one of the nine vaccine type HPV, and MSM were two and half times more likely to be seropositivity to ≥1 HPV types, indicating the need for HPV immunization in men, and particularly among MSM.

### Conclusions

Overall, exposure to 9vHPV types was common among men. Seropositivity to 9vHPV vaccine types was associated with older age and the lifetime number of anal sex partners. HPV vaccine could protect against HPV infection, and will reduce the burden of HPV related diseases among men.
